# A multicenter phase III trial comparing irinotecan-gemcitabine (IG) with gemcitabine (G) monotherapy as first-line treatment in patients with locally advanced or metastatic pancreatic cancer

**DOI:** 10.1038/sj.bjc.6603301

**Published:** 2006-08-08

**Authors:** G P Stathopoulos, K Syrigos, G Aravantinos, A Polyzos, P Papakotoulas, G Fountzilas, A Potamianou, N Ziras, J Boukovinas, J Varthalitis, N Androulakis, A Kotsakis, G Samonis, V Georgoulias

**Affiliations:** 1 Hellenic Oncology Research Group (HORG) and Hellenic Cooperative Oncology Group (HeCOG), Semitelou 2A, 115 28 Athens, Greece

**Keywords:** irinotecan, gemcitabine, pancreatic cancer

## Abstract

Our purpose was to determine the response rate and median and overall survival of gemcitabine as monotherapy versus gemcitabine plus irinotecan in advanced or metastatic pancreatic cancer. Patients with histologically or cytologically confirmed adenocarcinoma who were chemotherapy and radiotherapy naive were enrolled. Patients were centrally randomised at a one-to-one ratio to receive either gemcitabine monotherapy (900 mg m^−2^ on days 1, 8 and 15 every 4 weeks (arm G), or gemcitabine (days 1 and 8) plus irinotecan (300 mg m^−2^ on day 8) (arm IG), repeated every 3 weeks. The total number of cycles administered was 255 in the IG arm and 245 in the G arm; the median number of cycles was 3. In all, 145 patients (71 in arm IG and 74 in arm G) were enrolled; 60 and 70 patients from arms IG and G, respectively, were evaluable. A complete clinical response was achieved in three (4.3%) arm G patients; nine (15%) patients in arm IG and four (5.7%) in arm G achieved a partial response. The overall response rate was: arm IG 15% and arm G 10% (95% CI 5.96–24.04 and 95% CI 2.97–17.03, respectively; *P*=0.387). The median time to tumour progression was 2.8 months and 2.9 months and median survival time was 6.4 and 6.5 months for the IG and G arms, respectively. One-year survival was 24.3% for the IG arm and 21.8% for the G arm. No statistically significant difference was observed comparing gemcitabine monotherapy versus gemcitabine plus irinotecan in the treatment of advanced pancreatic cancer, with respect to overall and 1-year survival.

Owing to the nonspecific symptoms of the disease or to its insidious evolution, pancreatic cancer is often diagnosed when the disease is at an advanced stage; as a result, fewer than 20% of newly diagnosed patients are able to have a radical excision of the tumour (Brenna *et al*, 1993; Schnall and Macdonald, 1996; Stephens, 1998). Chemotherapy is the treatment of choice in patients with locally advanced and metastatic disease. Gemcitabine as a single agent is considered to be the standard treatment for these patients since, in spite of the low overall objective response, studies have shown an improvement in overall survival and a statistically significant clinical benefit when compared to the best supportive care (Casper *et al*, 1994; Burris *et al*, 1997).

Other single agents have also been tested in advanced pancreatic cancer but the response rate and survival have remained low; moreover, the incidence of clinical benefit obtained with these drugs has been variable (Wagener *et al*, 1995; Scher *et al*, 1996; Androulakis *et al*, 1999; Rougier *et al*, 2000; Konstandoulakis *et al*, 2001; Cartwright *et al*, 2002). Several phase II studies have evaluated different combinations of cytotoxic agents in order to improve the proportion of objective responses and the duration of survival. Numerous phase II studies have investigated combinations of these active drugs with or without gemcitabine in patients with advanced/metastatic pancreatic cancer (Rothenberg *et al*, 1998; Bahadori *et al*, 1999; Hidalgo *et al*, 1999; Rocha-Lima *et al*, 1999; Heinemann *et al*, 2000; Stathopoulos *et al*, 2001; Alberts *et al*, 2002; Berlin *et al*, 2002; Hess *et al*, 2003; Stathopoulos *et al*, 2003; Ulrich-Pur *et al*, 2003). The combination of gemcitabine plus irinotecan has resulted in an objective response of 25% with a median overall survival ranging from 5.7 to 7 months (Rocha-Lima *et al*, 2002; Stathopoulos *et al*, 2004). As phase II studies of combinations of active anticancer drugs in patients with advanced/metastatic pancreatic cancer have been associated with a better survival (about 7 months) (Rocha-Lima *et al*, 2002; Stathopoulos *et al*, 2003; Stathopoulos *et al*, 2004) compared with gemcitabine monotherapy (about 5 months) (Miller *et al*, 1981), various randomised trials are ongoing in order to validate these observations.

Despite the fact that the achieved objective response rate with gemcitabine-based combinations is practically similar, there are controversial results concerning overall survival; the 1-year survival was reported to be 22% with the gemcitabine–irinotecan combination (objective response rate (ORR) 25%) whereas overall survival was found to be 34.8% with the gemcitabine–capecitabine combination (ORR 18.9%) (Stathopoulos *et al*, 2003; Ulrich-Pur *et al*, 2003).

The Gastrointestinal Working Parties of the Hellenic Oncology Research Group (HORG) and the Hellenic Cooperative Oncology Group (HeCOG) conducted this intergroup, multicenter, phase III randomised trial in order to evaluate the efficacy of the gemcitabine-irinotecan combination versus gemcitabine monotherapy, in previously untreated patients with inoperable locally advanced or metastatic pancreatic cancer.

## PATIENTS AND METHODS

Patients >18 years of age with histologically or cytologically confirmed adenocarcinoma of the pancreas and bidimensionally measurable disease, who were chemotherapy and radiotherapy naive were enrolled in the study. Other eligibility criteria included a World Health Organization (WHO) performance status (PS) of 0–2, a life expectancy of at least 3 months, an adequate bone marrow reserve (granulocyte count ⩾1500 dl^−1^, platelet count ⩾120 000 dl^−1^), adequate renal (serum creatinine concentration <1.2 mg dl^−1^) and liver function (total serum bilirubin concentration <3 mg dl^−1^) provided that serum transaminases and serum proteins were normal; normal cardiac function with no history of clinically unstable angina pectoris or myocardial infraction or congestive heart failure within the 6 months prior and no central nervous system involvement. Prior surgery was allowed provided that it had taken place at least 3 weeks before enrollment. Patients with active infection, malnutrition or a second primary tumour (except for a nonmelanoma skin epithelioma or *in situ* cervix carcinoma) were excluded from the study. All patients gave their written informed consent to participate in the study.

### Treatment

All patients were treated on an outpatient basis. Patients were centrally randomised by computer at a one-to-one ratio to receive either monotherapy (arm G) with gemcitabine (Gemzar; Eli Lilly, Indianapolis, IN, USA) at a dose of 900 mg m^−2^ as a 60 min i.v. infusion on days 1, 8 and 15 every 4 weeks or the combination (arm IG) of gemcitabine (same dose on days 1 and 8) plus irinotecan (Campto; Sanofi-Aventis Collegeville, PA, USA) on day 8 at a dose of 300 mg m^−2^ over a 90 min i.v. infusion every 3 weeks. Cycles were continued provided that patients had sufficiently recovered from drug-related side effects. The allocation to either regimen was done by stratified randomisation according to age, performance status and stage of the disease. Standard antiemetic treatment with ondansetron was administered to all patients. Prophylactic recombinant human granulocyte colony-stimulating factor (rhG-CSF; Granocyte, Sanofi-Aventis) was allowed only in patients with ⩾grade 3 granulocytopenia and given at a dose of 150 *μ*g m^−2^ subcutaneously. Patients with an objective response or stable disease received at least six chemotherapy cycles. The protocol was approved by the Ethics and Scientific Committees of the participating hospitals.

Dose adjustment criteria were based on haematological parameters. Irinotecan and gemcitabine doses were reduced by 25% in cases of febrile or grade 4 neutropenia or thrombocytopenia. In cases of grade 3 neutropenia and/or thrombocytopenia lasting for >5 days, the dose of both drugs was reduced by 15%. Toxicities were graded according to WHO guidelines (Miller *et al*, 1981).

### Evaluation of patients

Pretreatment evaluation included a complete medical history and physical examination, a full blood cell count with differential and platelet count, a standard biochemical profile, serum carcinoembryonic antigen (CEA) and CA 19-9 determinations, electrocardiogram, chest X-rays, ultrasound of the upper abdomen and computed tomography scans of the chest and upper and lower abdomen. Additional imaging studies were performed on clinical indication; these studies were performed and analysed by the same radiologist. Full blood cell counts with differential were performed weekly; in cases of grade 3–4 neutropenia or grade 4 thrombocytopenia, full blood cell counts with differential were evaluated daily until the absolute granulocyte count was ⩾1000 dl^−1^ and the platelet count ⩾75 000 dl^−1^. A detailed medical and physical examination was performed before each course of treatment in order to document disease symptoms and treatment toxicity. Biochemical tests, electrocardiogram (ECG), serum CEA and CA 19-9 determinations and chest X-rays were performed every 6 weeks. A neurological evaluation was performed by clinical examination every 6 weeks. Lesions were measured after each cycle if they were assessable by physical examination or by chest X-rays; lesions assessable by ultrasound or CT scan were measured after 3 cycles of chemotherapy.

### Definition of response

A complete response (CR) was defined as the disappearance of all measurable or evaluable disease, signs and symptoms and biochemical changes related to the tumour for at least 4 weeks, during which time no new lesions may appear; partial response (PR), a>50% reduction in the sum of the products of the perpendicular diameters of all measurable lesions compared with pretreatment measurements lasting ⩾24 weeks, during which time no new lesions may appear and no existing lesions may enlarge. For hepatic lesions, a reduction of >30% in the sum of the measured distances from a costal margin at the midclavicular line and at the xiphoid process to the edge of the liver was required. Stable disease (s.d.) was defined as a <50% reduction or a <25% increase in the sum of the products of the two perpendicular diameters of all measured lesions and the appearance of no new lesions for 8 weeks. Progression or relapse was defined as an increase in the product of the two perpendicular diameters of any measurable lesion by >25% over the size present at enrolment or for patients who responded, the size at the time of maximum regression and the appearance of new areas of malignant disease (usually excluding central nervous system metastases). A deterioration in performance status, loss of >10% pretreatment weight or worsening symptoms did not by themselves constitute progression; however, persistence of these complaints or the appearance of new symptoms required a repeat evaluation of the extent of the disease (Donehower *et al*, 1995). All responses had to be maintained for ⩾4 weeks and had to be confirmed by an independent panel of radiologists.

### Assessment of clinical benefit

The assessment of pain was based on both the consumption of analgesics (narcotics and non-narcotics) and the patient's own evaluation using a scale graded from 0 (no pain) to 10 (maximum pain necessitating narcotics for relief). A >50% decrease of analgesic consumption with no need for narcotics coupled with the patient's evaluation of a >50% decrease in pain intensity was characterised as ‘pain improvement.’ A >50% increase in the consumption of analgesics in combination with the patient's evaluation or a >50% increase in pain intensity was characterised as ‘pain deterioration.’ All other cases were characterised as ‘no change.’ Symptoms of vomiting and diarrhoea were assessed according to the number of daily episodes: a 50% decrease in number was characterised as ‘improvement’ whereas a >50% increase, as ‘deterioration.’ All other cases were characterised as ‘no change.’ In addition, patients were asked to grade their fatigue and anorexia using a scale of 0 (no fatigue or anorexia) to 10 (maximum fatigue or anorexia). A 50% decrease or increase in symptom intensity indicated ‘improvement’ or ‘deterioration,’ respectively.

### Statistical analysis

The study was designed as a group-sequential clinical trial. An interim analysis based on the O’Brien/Fleming boundary values was performed when 50% of the endpoints had been reached (Rocha-Lima *et al*, 2004). The study would have ended prematurely if a significant difference in survival had been observed. The randomisation of patients into two treatment arms was performed according to the method of random permuted blocks within strata. Stratification factors comprised stage III or and IV disease. Dynamic balancing was performed by the hospital. Pearson's *χ*^2^ test (or Fisher's exact test when appropriate) was used for the comparisons of categorical variables. The nonparametric Mann–Whitney test was used for comparisons of continuous variables. Time-to-event analyses were performed where survival distribution was estimated by the Kaplan–Meier curve, and treatment comparison was made using the log-rank test. All reported *P*-values are two-sided. A *P*-value of <0.05 was considered significant. The primary end point was median survival time and the secondary end points were response rate, median time to tumour progression and tolerance.

The randomisation was carried out at the University Hospital of Heraklion, Crete, in the Office of Clinical Trials. There were eight losses to follow-up (four per treatment group). The analysis of data was done on an intention-to-treat analysis basis. The accrual time was 36 months and the median follow-up time, 24 months.

## RESULTS

### Patient demographics

From November 2001 to February 2005, 145 patients (71 in the IG arm and 74 in the G arm) were enrolled. Eleven (15.5%) IG arm and 4 (5.4%) G arm patients were not evaluated for the following reasons: arm IG: a PS of 3 in three patients, protocol violation in two, consent withdrawn by six patients; arm G: renal failure in one patient, no measurable disease in one patient, protocol violation in one, and consent withdrawn by one patient. The patients' characteristics are shown in Table 1. The median age was 64 years in each arm and 78% arm IG and 86% arm G patients had stage IV disease. Although the difference in stage IV patients enrolled in the IG and G arms was about 8%, this difference was not statistically significant (*P*=0.272). The median number of involved organs was 2 (range, 1–4) in each arm; liver involvement was present in 34% and 37% IG and G patients, respectively; similarly, 10 and 11% patients enrolled in the IG and G arms, respectively, had abdominal lymph node involvement.

### Compliance with treatment

The total number of administered chemotherapy cycles were 255 (median 3; range, 1–16) for patients treated with the IG regimen and 245 (median 3; range 1–8) for those treated with the G regimen. The median interval between cycles was 21 (range, 21–30.8) and 29 days (range 28–35) for the IG and G arms, respectively. The median dose intensity was 83 mg m^−2^ week^−1^ (range, 25–100) for irinotecan and 553 mg m^−2^ week^−1^ (range, 299–600) for gemcitabine (IG arm) and 591 mg m^−2^ week^−1^ (range, 179–675) for gemcitabine (G arm). Twelve (4.7%) of the IG arm and 13 (5.3%) of the G arm cycles were delayed. The reasons for treatment delay were: in arm IG, haematologic toxicity in five patients, nonhaematologic toxicity in one patient, and nonrelated to treatment or the disease (non-neutropenic infections) in six patients; in arm G, haematologic toxicity in four patients, nonhaematologic toxicity in two patients and nonrelated to treatment or the disease in six patients. Dose reduction was required in eight (13.3%) patients treated with IG and 17 (24.3%) patients treated with G (*P*<0.001). Dose reduction was required in 34 (13.3%) of the IG arm and 60 (24.5%) of the G arm cycles. Haematologic toxicity was the main reason for dose reduction in both groups; however, the incidence of haematologic toxicity was higher (35.3%) in patients treated with IG than in those treated with G (26.7%) (*P*<0.001) Table 2. In addition, the incidence of no drug administration on day 8 and/or day 15 was higher (46.7%) in patients enrolled in the G arm versus 29.4% in the IG arm (*P*=0.002). Treatment was completed as per protocol in 18 (30%) IG arm patients and in 12 (17.1%) G arm patients whereas it was stopped because of disease progression in 29 (48.3%) IG arm patients and in 49 (70.%) G arm (*P*=0.027).

The total number of deaths at the end of the study were 46 (76.7%) and 57 (81.4%) patients enrolled in the IG and G arms, respectively. Death was due to malignant disease in 84.8% and 84.2% of the G and IG patients, respectively. One patient in arm G died because of stroke.

### Response to treatment

Responses were analysed on an intention-to-treat basis. There were no complete responses in the IG-arm patients; however, a complete clinical response was achieved in three (4.3%) patients treated with G. In addition, nine (15%) IG arm and four (5.7%) G arm patients achieved a partial response (overall response rate: IG arm, 15% and G arm 10%, 95% CI 5.96–24.04% and 2.97–17.03, respectively; *P*=0.387). Stable disease was achieved in 16 (26.7%) IG arm and 13 (18.6%) G arm patients, while disease progression was observed in 35 (58.3%) and 50 (71.4%) of the IG and G arm patients, respectively. Tumour disease control (CR+PR+SD) was achieved in 25 (41.7%; 95% CI: 29.19–54.14) and in 20 (28.6%; 95% CI 17.99–39.15) patients treated with IG and G, respectively, (*P*=0.800). The median time to tumour progression (TTP) was 2.8 months (range 1.0–17.3 months) and 2.9 months (range 1.0–17.4 months) for patients treated with IG and G, respectively (*P*=0.795).

**Figure 1:**
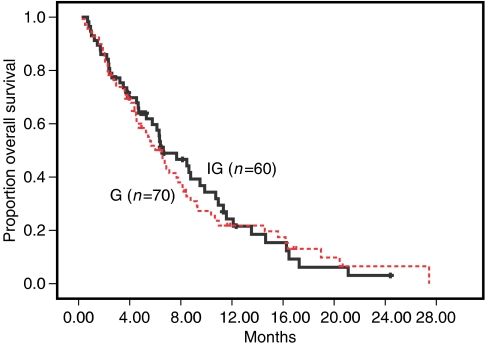
Kaplan–Meier overall survival.

After a median follow-up period 5.9 months (range, 1.0–24.4 months) for IG arm patients and 5.3 months (range, 1.0–27.4) for G arm patients, 46 (76.7%) and 57 (81.4%) patients, respectively, died. The median survival time was 6.4 months (range, 1.0–24.4 months) and 6.5 months (range, 1.0–27.4 months) in patients treated with IG and G, respectively (*P*=0.970). The 1-year survival was 24.3% in patients treated with IG and 21.8% in patients treated with G (Figure 1). In all, 21 (35%) in the IG and 22 (31.4%) in the G group received second-line chemotherapy. The difference was not statistically significant (*P*=0.666) with regard to survival benefit.

### Effect of treatment on serum levels of CA 19-9

In 52 (86.7%) and 60 (85.7%) patients treated with the IG and G regimens, respectively, there were sufficient evaluable data on the serum levels of CA 19-9. In 24 (46.2%) IG arm and in 22 (36.7%) G arm patients, a >25% decrease of serum levels of CA 19-9 was observed (*P*=0.308). Similarly, 13 (25%) and 23 (38.3%) patients treated with the IG and G regimens, respectively, showed a >25% increase in the serum levels of CA 19-9 during treatment. There was no clear correlation between CA 19-9 measurements and radiological response (*P*=0.226).

### Effect of treatment on tumour-related symptoms

Tumour-related symptoms were present at enrolment in 48 (80%) and 58 (82.9%) of patients treated with IG and G, respectively. There was no significant difference between the two chemotherapy regimens with regard to their effect on tumour-related symptoms.

### Toxicity

Table 3 shows the incidence of severe (grade 3 and 4) haematologic and non-haematologic toxicity associated with the IG and G regimens. Grade 3 and 4 neutropenia occurred in 16 (26.7%) patients treated with IG and 11 (15.7%) patients treated with G (*P*=0.125). No patients developed febrile neutropenia. Grade 3 and 4 thrombocytopenia occurred in three (5.0%) patients treated with IG (*P*=0.028). Nonhaematologic toxicity was mild, usually <5%, regardless of the chemotherapy regimen administered.

## DISCUSSION

Locally advanced or metastatic pancreatic cancer is an incurable disease and in this setting, only systemic chemotherapy may result in a small improvement in survival and clinical benefit. In an effort to improve the results obtained with gemcitabine, which is the standard treatment, several phase II studies have evaluated it in combination with other cytotoxic agents. Some of these studies have reported an improved median overall survival and 1-year survival rates. However, the question which concerns the superiority of gemcitabine-based chemotherapy regimens over gemcitabine monotherapy in terms of overall survival and quality of life can only be answered by comparative randomised studies.

The present study was based on the promising results of a previous phase II trial of irinotecan and gemcitabine in patients with advanced/metastatic pancreatic cancer, which reported an objective response rate of 25% (Stathopoulos *et al*, 2003). This efficacy of the irinotecan-gemcitabine combination was higher than that obtained with gemcitabine alone. Similar results have also been reported with the irinotecan-gemcitabine regimen by another research group (Rocha-Lima *et al*, 2002). The results of the present study demonstrate that although the ORR was higher with the IG regimen compared with that of the G regimen, this difference did not reach statistical significance. Moreover, there was no difference between the two treatment arms in terms of duration of response, TTP, overall survival and 1-year survival.

The results of a randomised trial comparing the irinotecan–gemcitabine combination with gemcitabine alone in patients with locally advanced and metastatic pancreatic cancer have recently been reported (Rocha-Lima *et al*, 2004). This study demonstrated a significantly higher ORR of 16.1% with the combination versus that achieved with gemcitabine alone (ORR 4.4%); moreover, the ORR was also higher with the two-drug combination (25.9%) compared with gemcitabine monotherapy (ORR 4.2%). In addition, these authors (Rocha-Lima *et al*, 2004) reported that the TTP was significantly higher in patients with locally advanced disease treated with the irinotecan–gemcitabine combination compared with gemcitabine as a single agent. However, there was no difference between the two chemotherapy regimens in terms of overall and 1-year survival (Rocha-Lima *et al*, 2004). It is interesting to note that the above study (Rocha-Lima *et al*, 2004) and the present trial have documented a similar efficacy achieved with the irinotecan–gemcitabine combination; however, gemcitabine monotherapy resulted in a relatively low response rate (Rocha-Lima *et al*, 2004) which may account for the observed statistical difference.

These results were obtained with an acceptable toxicity profile for both regimens. Indeed, the incidence of severe grade 3 and 4 toxicity was practically similar in the two arms and only the incidence of severe thrombocytopenia was shown to be statistically higher in the combination arm compared with gemcitabine monotherapy. In addition, the incidence of severe asthenia was higher in the monotherapy arm compared to the combination arm.

It is difficult to explain why the combinations of gemcitabine with a second anticancer drug cannot significantly improve the overall survival of patients with locally advanced/metastatic pancreatic cancer. This may reflect the natural history of the tumour, which is characterised by its indolent onset and its inoperability at the time of diagnosis. The clinical characteristics of this disease are directly related to the biology of the tumour cell. Therapeutic approaches which target the specific biologic mechanisms involved in tumour cell proliferation and metastasis might be revealed to be more effective in these patients. The recently reported improvement of overall survival in patients with advanced and metastatic pancreatic cancer with the gemcitabine–erlotinib combination compared to single-agent gemcitabine supports this hypothesis (Moore *et al*, 2005). Additional studies evaluating novel agents against specific molecular targets alone or in combination with other cytotoxic agents are necessary.

## Figures and Tables

**Table 1:** Patients' characteristics

	**Treatment groups**	
	**IG (*n*=60)**	**G (*n*=70)**
	***n* (%)**	***n* (%)**
Age (year)		
Median (range)	64 (31–84)	64 (44–83)
		
*Gender*		
Male	39 (65)	42 (60)
Female	21 (35)	28 (40)
		
*Performance status (WHO)*		
0–1	52 (87)	60 (86)
2	8 (13)	10 (14)
		
*Stage*		
III	13 (22)	10 (14)
IV	47 (78)	60 (86)
		(*P*=0.272)
		
*No. of organs involved*		
1	24 (40)	24 (34)
2	22 (37)	34 (49)
>3	14 (23)	12 (17)
		
Prior Surgery	11 (18)	16 (23)
		
No prior treatment	49 (82)	54 (77)

**Table 2:** Reasons for dose reduction

	**Treatment groups**	
	**IG (255 cycles)**	**G (245 cycles)**
	***n* (%)**	***n* (%)**
Dose reduction	34 (13.3)	60 (24.5)
Due to:		
Haematologic toxicity	12 (35.3)*	16 (26.7)
Nonhaematologic toxicity	10 (29.4)	8 (13.3)
Haematologic and nonhaematologic toxicity	1 (2.9)	1 (1.7)
Day 8 and/or day 15 treatment not given (for reasons other than toxicity)	10 (29.4)	28 (46.7)†
		
Other	1 (2.9)	7 (11.7)

* *P*<0.001.

† *P* 0.002.

**Table 3:** Severe (grade 3 and 4) haematologic and nonhaematologic toxicity

	**Grade 3 and 4 (WHO)**		
	**IG**	**G**	
	***n*=60**	***n*=70**	
	***n* (%)**	***n* (%)**	***P*-value**
Anemia	3 (5)	3 (4.3)	NS
Neutropenia	16 (26.7)	11 (15.7)	0.125
Thrombocytopenia	3 (5)	—	0.028
Nausea	1 (1.7)	2 (2.9)	NS
Vomiting	1 (1.7)	1 (1.4)	NS
Diarrhea	2 (3.3)	2 (2.9)	NS
Asthenia	—	4 (5.7)	—
Influenza-like syndrome	2 (3.3)	—	
